# scEGG: an exogenous gene-guided clustering method for single-cell transcriptomic data

**DOI:** 10.1093/bib/bbae483

**Published:** 2024-09-29

**Authors:** Dayu Hu, Renxiang Guan, Ke Liang, Hao Yu, Hao Quan, Yawei Zhao, Xinwang Liu, Kunlun He

**Affiliations:** School of Computer, National University of Defense Technology, No. 109 Deya Road, 410073 Changsha, Hunan, China; School of Computer, National University of Defense Technology, No. 109 Deya Road, 410073 Changsha, Hunan, China; School of Computer, National University of Defense Technology, No. 109 Deya Road, 410073 Changsha, Hunan, China; School of Computer, National University of Defense Technology, No. 109 Deya Road, 410073 Changsha, Hunan, China; College of Medicine and Biological Information Engineering, Northeastern University, No.195 Chuangxin Road, 110169 Shenyang, Liaoning, China; Medical Big Data Research Center, Chinese PLA General Hospital, No. 28 Fuxing Road, 100853 Beijing, China; School of Computer, National University of Defense Technology, No. 109 Deya Road, 410073 Changsha, Hunan, China; Medical Big Data Research Center, Chinese PLA General Hospital, No. 28 Fuxing Road, 100853 Beijing, China

**Keywords:** exogenous gene information, clustering, protein-protein interaction, Node2vec, deep learning

## Abstract

In recent years, there has been significant advancement in the field of single-cell data analysis, particularly in the development of clustering methods. Despite these advancements, most algorithms continue to focus primarily on analyzing the provided single-cell matrix data. However, within medical contexts, single-cell data often encompasses a wealth of exogenous information, such as gene networks. Overlooking this aspect could result in information loss and produce clustering outcomes lacking significant clinical relevance. To address this limitation, we introduce an innovative deep clustering method for single-cell data that leverages exogenous gene information to generate discriminative cell representations. Specifically, an attention-enhanced graph autoencoder has been developed to efficiently capture topological signal patterns among cells. Concurrently, a random walk on an exogenous protein–protein interaction network enabled the acquisition of the gene’s embeddings. Ultimately, the clustering process entailed integrating and reconstructing gene-cell cooperative embeddings, which yielded a discriminative representation. Extensive experiments have demonstrated the effectiveness of the proposed method. This research provides enhanced insights into the characteristics of cells, thus laying the foundation for the early diagnosis and treatment of diseases. The datasets and code can be publicly accessed in the repository at https://github.com/DayuHuu/scEGG.

## Introduction

Single-cell transcriptome sequencing technology represents a significant advancement in the field of genomics. It elucidates the intricate biological processes at the cellular level and serves as a potent tool for studying the origins and microenvironments of tumors [1–6]. Unsupervised clustering represents a pivotal step in this process. By analyzing the gene expression data of individual cells, it precisely differentiates between various cell types and states. This approach provides valuable insights into understanding complex biological systems, such as cancer, neurodegenerative diseases, and developmental processes. However, owing to the complexity of biological systems, devising a clustering algorithm that is both accurate and highly clinically relevant remains a formidable challenge.

In recent years, a significant increase in the development of clustering algorithms tailored for single-cell RNA sequencing (scRNA-seq) data has been observed [7–10]. Early approaches depended on probabilistic models that estimated high-dimensional cell data through computing the probability of gene expression. For instance, CIDR [11] introduced an interpolation method to handle dropout events, whereas SC3 [12] employed hierarchical $k$-means clustering to facilitate consensus clustering, presuming Euclidean relationships between cells. However, these methods operate under the assumption that biological data are linear and devoid of noise, an assumption that is often not valid in practical scenarios.

To effectively extract features from scRNA-seq data and circumvent assumptions about data distribution, some researchers have suggested neural networks as a promising approach for mining information from scRNA-seq data [13–16]. Neural networks, widely used as black box models, can adapt to nearly all data distributions when the parameters are appropriately configured. Numerous single-cell deep clustering algorithms have been proposed to obtain effective representations. However, these models often treat cells as isolated entities, overlooking the associations between them. To integrate cellular interaction relationships into the clustering process, researchers have proposed graph-based approaches for deriving cell embeddings. This approach necessitates constructing a cell graph based on intercellular similarities. These constructed graphs, in conjunction with the original feature matrix, are subsequently inputted into a graph neural network for training. A detailed introduction to these methods will be provided in the Related Work section (Section 4). Although these graph-based deep clustering algorithms have progressed in capturing the topological features of cells, their focus remains primarily on analyzing the provided single-cell matrix data. However, clustering algorithms oriented towards medical applications should integrate external information for a more holistic analysis, as overlooking this aspect could result in clustering outcomes that diverge from clinical conclusions.

**Figure 1:**
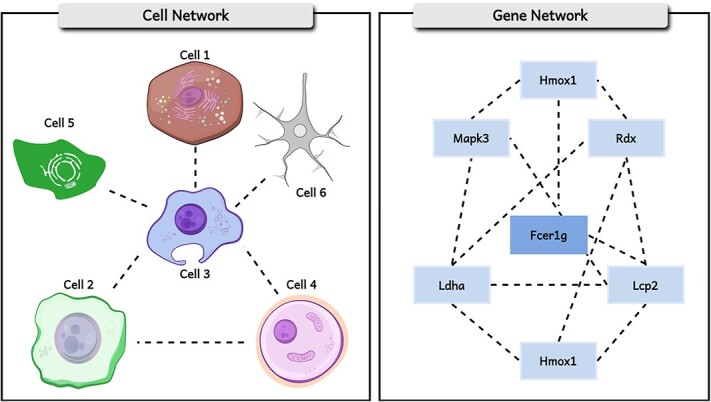
Cells and genes both exhibit associative relationships. The left illustrates the connections between cells, while the right depicts the associations among genes. The cell image originates from the SciDraw database.

Single-cell data inherently contain exogenous information. Unlike other datasets, the features in scRNA datasets are meaningful as they represent genes. Biologists and medical scientists have extensively explored gene relationships. Despite this extensive research, most current clustering methods still overlook these gene relationships, focusing solely on cell connections. However, in reality, genes within each cell participate in complex interrelations due to interactions, regulatory mechanisms, and shared functions and pathways in biological processes. In essence, as shown in Fig. 1, gene topological features are evident. These features might be omitted as researchers are concerned that introducing prior knowledge could inadvertently disclose information about cell identity, thus compromising the unsupervised nature of the clustering model. However, the gene graph merely transforms the original features, thereby circumventing the aforementioned issue of information disclosure. The primary challenges in incorporating exogenous genes into the existing clustering framework are two-fold: (1) accomplishing the conversion from genes to embeddings; (2) ensuring that no truncation of features occurs during this transformation process. Some pioneering works study the conversion of genes into embeddings. A detailed review to these methods will be provided in Section 4. However, this aspect remains largely unexplored in single-cell clustering research. By extracting and integrating the topological features of gene interconnectivity into the clustering framework, significant optimization of clustering embeddings and enhancement of clustering outcomes can be achieved. Furthermore, this type of embedding could lead to a more accurate representation of biological characteristics, thereby enhancing the alignment between identified clusters and the actual underlying biological systems.

In light of these considerations, we have developed an Exogenous Gene-Guided single-cell deep clustering method (scEGG) that focuses on the gene-cell cooperative embedding. To accomplish this, we utilized a graph attention autoencoder (GAT), which captures the topological structure between cells and ensures effective information transmission among them. Additionally, we conducted random walks on the exogenous protein–protein interaction (PPI) network corresponding to the gene set to obtain embeddings that represent the gene’s topological features. During the clustering process, we integrated these two elements and reconstructed the features of gene-cell cooperative embedding, thereby acquiring a discriminative cell representation. Experiments on six real scRNA datasets demonstrate that our scEGG method is stable and outperforms eight other baseline methods. Our contributions can be summarized as follows:

- We pioneered an exogenous gene-guided clustering framework that generates gene-cell cooperative embeddings and learns a more discriminative representation through optimization. This work establishes a paradigm for integrating exogenous medical information into the clustering process.
- The proposed scEGG model employs a GAT to accurately aggregate information among cells and uses reconstruction loss and clustering loss to facilitate the optimization of the bottleneck layer, effectively utilizing its own information without requiring labels.
- The experiment demonstrates the effectiveness and superior performance of scEGG when compared to the other eight baseline methods.

## Related work

### Single-cell deep clustering

Recently, deep learning methods have been widely applied to analyze scRNA-seq data due to their formidable learning capabilities. Li *et al*. proposed DESC, which iteratively learns the gene expression pattern of each cluster, assigns cells to their respective clusters and continuously mitigates batch effects [17]. Tian *et al*. propose scDeepCluster [18], a method rooted in the Zero-inflated Negative Binomial (ZINB) model, utilizing a bottleneck layer for deep $k$-means clustering to enhance clustering outcomes. Tian *et al*. developed a deep embedding clustering approach for single-cell data, integrating the ZINB model with clustering loss and constraint loss [19]. However, these deep neural networks struggle to preserve the topological structure of scRNA-seq data, because they neglect the associations between cells during analysis. The advent of deep graph autoencoders has addressed the aforementioned concerns, namely, that previous models treated cells as isolated individuals. These graph autoencoders efficiently learn cluster-friendly, low-dimensional representations by incorporating graph topology information of cell-to-cell interactions. Satija *et al*. proposed Seurat [20], which employs Louvain community detection to construct a cell graph, subsequently analyzed through spectral clustering using Phenograph. Wang *et al*. proposed scGNN [21], which utilizes a graph neural network to capture and integrate relationships between cells, complemented by a Gaussian model to represent the pattern of heterogeneous gene expression. Yu *et al*. introduced scTAG [22], a specialized deep graph embedding clustering algorithm tailored for single-cell data, which concurrently optimizes clustering loss, ZINB loss, and cell graph reconstruction loss. Furthermore, Chen proposed scGAC [23], which introduces attention mechanisms based on the cell-to-cell graph, thus ensuring effective information transmission between cells. Meanwhile, our previous model, scDFC [24], combines structural data from cell-to-cell graphs with attribute information from cellular expression patterns, thereby facilitating a comprehensive analysis of scRNA data.

### The conversion of genes to embeddings

The premise of incorporating genes into a deep learning framework is that genes are learnable, which necessitates the transformation of genes from characters to numerical values. Recently, some studies have explored this aspect. Gene2vec adapts word embedding techniques from natural language processing to biomedical applications [25], thereby generating vector representations for genes. Similarly, Woloszynek *et al*. employ the Skip-Gram word2vec model for nucleotide sequences [26], while fastDNA maps DNA sequences into vector spaces [27], utilizing low-dimensional representations of k-mers. Moreover, BERT-RBP utilizes the Bidirectional Encoder Representations from Transformers model to encode the human reference genome [28], thus facilitating the prediction of RNA–protein interactions. Although effective, these vector transformation methods tailored for genes often overlook the interactions between genes. Therefore, in this study, we propose adopting the random walk method from the field of graph representation learning to the biological domain to generate graph representations for genes.

## Preliminaries

Single-cell data refers to genetic expression information obtained through single-cell sequencing technology, which is presented in matrix form. In this work, we provide a simple mathematical description of this data, which is represented as a numerical matrix denoted by $\mathbf{X}^{(c)}\in \mathbb{R}^{n \times d_{g}}$, where $d_{g}$ denotes the number of genes, and $n$ represents the number of cells. The superscripts (c) and (g) denote ’cell’ and ’gene’, respectively. $X^{(c)}_{ab}$ denotes the expression count of the $b$th highly variable gene in the $a$th cell. Consistent with many methods, we use the Pearson correlation coefficient to calculate the correlation between cells. The calculation process is detailed below: 


(1) \begin{align*}& P_{ij} = \frac{\sum_{k=1}^{d} ({X}^{(c)}_{ik} - \overline{{X}^{(c)}_{i}})({X}^{(c)}_{jk} - \overline{{X}^{(c)}_{j}})}{\sqrt{\sum_{k=1}^{d} ({X}^{(c)}_{ik} - \overline{{X}^{(c)}_{i}})^{2} \sum_{k=1}^{d} ({X}^{(c)}_{jk} - \overline{{X}^{(c)}_{j}})^{2}}},\end{align*}

In the given raw data, ${X}^{(c)}_{ik}$ and ${X}^{(c)}_{jk}$ represent the elements in the $k$th column of rows $i$ and $j$, respectively, with $ \overline{{X}^{(c)}_{i}}$ and $ \overline{{X}^{(c)}_{j}}$ as their respective row means. We utilize the K-nearest neighbors (KNN) algorithm to construct the cell graph $\mathcal{G}^{(c)}$, where each node corresponds to a cell, leading to the adjacency matrix $\mathbf{A}^{(c)}$ for $\mathbf{X}^{(c)}$. The adjacency matrix $\mathbf{A}^{(c)}$ is defined as an $n \times n$ matrix, where each element $\mathbf{A}^{(c)}_{ij}$ represents the presence of an edge connecting node $i$ to node $j$, with 1 indicating an existing edge and 0 indicating the absence thereof.

## Methods

This section outlines the scEGG model, which performs representation learning and clustering on a preprocessed cell matrix through the following four sequential stages:

(a) **Deriving Gene Embeddings from Exogenous Gene Set:** utilizing the gene set derived from the dataset, input it into the official PPI network website to retrieve the corresponding gene graph. Following this, conduct a random walk on the graph to produce an initial representation for each gene.
(b) **Framework for Cooperative Training of Exogenous Genes and Cells**: The scEGG framework utilizes a graph attention network to aggregate inter-cellular information and generate cell embeddings. These are then element-wise multiplied with gene embeddings to produce gene-cell cooperative embeddings.
(c) **Optimization of Cell Representation:** subsequently, the obtained gene-cell cooperative embeddings are optimized by simultaneously minimizing both the reconstruction and clustering losses.
(d) **Generation of Clustering Results:** clustering is then performed on the learned cell representations.

### Deriving gene embeddings from exogenous gene set

Here we provide a comprehensive guide to constructing gene embeddings from single-cell datasets. Initially, each dataset is processed using Scanpy to identify highly variable genes. The top 500 genes are then uploaded to the online platform STRING (https://www.string-db.org/) to create a PPI network. Unlike previous methods, we utilize a random walk on the gene graph to generate graph embeddings.

**Figure 2:**
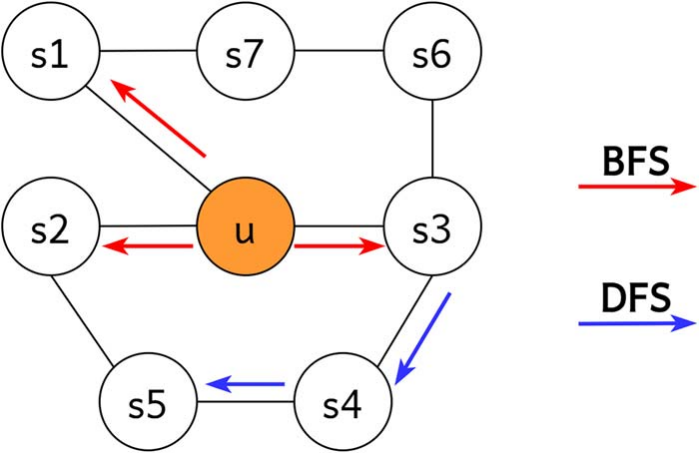
In BFS and DFS traversals, the node pointed to by the top line is considered a low-order neighbor of the source node $u$, while the node pointed to by the bottom line is considered a higher-order neighbor.

Various walking methods exist; to ensure generality and scalability, we employ node2vec to illustrate the random walk process. This biased walking strategy incorporates two neighborhood approaches: breadth-first (BFS) and depth-first (DFS) searches. BFS focuses on exploring nodes within the same layer, prioritizing traversal of nodes along red arrows. Meanwhile, DFS aims at higher-order nodes, prioritizing traversal of nodes along blue arrows. Figure 2 illustrates this random walk procedure.

Formally, consider $\mathcal{G}^{(g)}=(\mathcal{V},\mathcal{E})$ as a PPI network, with $\mathcal{V}$ representing the set of nodes, each corresponding to a protein sourced from a gene, and $\mathcal{E}$ indicating the interactions between these proteins. We utilize node2vec to generate an embedding vector for each node. Given a source node $u$, multiple random walk sequences are generated, each with a predetermined length of $M$. In each sequence $\{s_{1},s_{2},\dots ,s_{m}\}$, $s_{i}$ represents the $i$th node. To facilitate the transition from node $s_{i}$ to its neighboring node $v$, considering the preceding node $s_{i-1}$, we define the transition probability as follows: 


(2) \begin{align*}& p_{i,v}= \left\{ \begin{aligned} \frac{1}{p}, \quad \text{if}\ d_{(s_{i-1},v)}=0 \\ 1, \quad \text{if}\ d_{(s_{i-1},v)}=1\\ \frac{1}{q}, \quad \text{if}\ d_{(s_{i-1},v)}=2 \\ \end{aligned} \right.\end{align*}

where $d_{(s_{i-1}, v)}$ denotes the shortest path distance between nodes $s_{i-1}$ and $v$. The hyperparameters $p$ and $q$ are user-defined. A shortest path length of 0 indicates that node $v$ is the same as $s_{i-1}$, signifying a revisit. The parameter $p$ influences the likelihood of revisiting; a higher value reduces this probability. A shortest path length of 1 indicates that $v$ and $s_{i-1}$ are first-order neighbors, with neither BFS nor DFS strategies being applied. A shortest path length of 2 indicates that $v$ and $s_{i-1}$ are second-order neighbors. Here, $q$ influences the likelihood of moving towards more distant nodes: a higher $q$ biases the walk towards nodes closer to $s_{i-1}$, capturing a local view, while a lower $q$ encourages exploration further from $s_{i-1}$, facilitating DFS-like behavior. In brief, $p$ and $q$ adjust the probabilities in random walks for revisiting and extending exploration, thereby balancing breadth-first and depth-first walks to enhance effective sampling. Upon completing sequence sampling, we generate a corresponding representation for each node. For a given node $u$, our objective is to maximize the log probability of observing its neighboring nodes: 


(3) \begin{align*}& \begin{aligned} \max_{f}\sum_{u \in \mathcal{V}} \log Pr(N_{S}(u)|f(u)), \end{aligned}\end{align*}

here, $N_{S}(u)$ denotes the neighborhood of node $u$ according to the sampling strategy $S$. The function $f$ maps each node to its corresponding feature representation, where $f(u)$ represents the feature representation of node $u$. Optimizing Equation (3) produces embeddings for each node. We then concatenate these node vectors to obtain the gene embeddings: 


(4) \begin{align*}& \begin{aligned} \mathbf{X}^{(g)}=[f(u_{1}),f(u_{2}),\ldots,f(u_{d_{g}})]^{\top}, \end{aligned}\end{align*}

where $f(u_{i})$ denotes the embedding of the $i$th gene. It is important to note that the parameters of the node2vec algorithm in this study are set to their default values, as our goal is to prevent any influence on the performance of the scEGG model. 



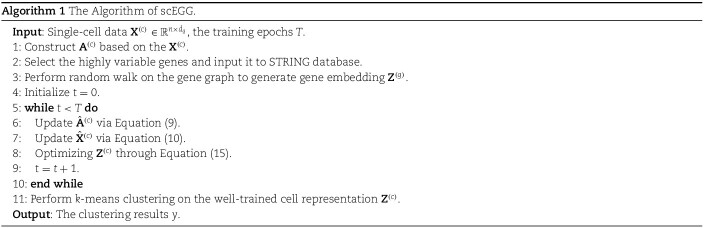



### Framework for cooperative training of exogenous genes and cells

**Figure 3:**
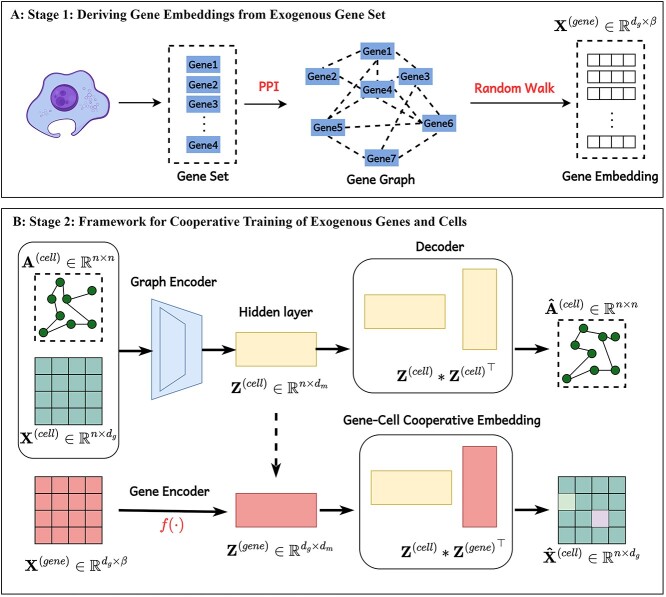
The scEGG model framework is divided into two stages. In stage 1, a random walk algorithm is applied to an exogenous gene network to generate distinct embeddings for each gene. In stage 2, the derived gene embeddings and cell embeddings are mapped to the same feature space, where they are integrated through matrix multiplication to construct a gene-cell cooperative embedding.

This section elucidates the methodologies employed in generating cell embeddings and gene-cell cooperative embeddings, as is shown in Fig. 3. Noise is commonly observed in single-cell data. To facilitate the precise transmission of information among cells, we developed a graph encoder enhanced with an attention mechanism that comprehensively captures cell signaling patterns and cell-to-cell relationships. The calculation process for the attention coefficient $\alpha _{ij}$ between nodes $i$ and $j$ is as follows: 


(5) \begin{align*}& \begin{aligned} \alpha_{ij} = \frac{\exp(\text{LeakyReLU}(\mathbf{a}^{T}[\mathbf{W}h_{i} \, \| \, \mathbf{W}h_{j}]))}{\sum_{k \in \mathcal{N}(i)} \exp(\text{LeakyReLU}(\mathbf{a}^{T}[\mathbf{W}h_{i} \, \| \, \mathbf{W}h_{k}]))}, \end{aligned}\end{align*}

where $\mathbf{a}$ is the learnable weight vector, $\mathbf{W}$ is the learnable weight matrix, and ∥ denotes the concatenation operation. $\mathcal{N}(i)$ denotes the neighbors of node $i$, while $h_{i}$ represents the feature vector of node $i$. Given the feature vector $h^{(l)}$ of the $l$th layer, the update formula for the next layer is 


(6) \begin{align*}& \begin{aligned} h_{i}^{(l+1)} = \sigma\left(\sum_{j \in \mathcal{N}(i)} \alpha_{ij} \mathbf{W} h_{j}^{(l)}\right), \end{aligned}\end{align*}

where $h_{i}^{(l+1)}$ denotes the feature vector at the next layer. To simplify computation process, we abbreviate the GAT calculation as follows: 


(7) \begin{align*}& \mathbf{Z}^{(c)} = \mathcal{F}_{e}^{c}(\mathbf{X}^{(c)}) \quad (\mathbf{Z}^{(c)} \in \mathbb{R}^{n \times d_{m}}),\end{align*}

where $\mathcal{F}_{e}^{c}$ denotes GAT mapping network, through which the original cell input is compressed into a low-dimensional feature space. For clarity, we append the dimensions of representations to the end of each equation. In a similar vein, we also devise a mapping network to effectuate feature transformation on the gene embeddings: 


(8) \begin{align*}& \begin{aligned} \mathbf{Z}^{(g)}=\mathcal{F}_{e}^{g}(\mathbf{X}^{(g)}) \quad (\mathbf{Z}^{(g)}\in\mathbb{R}^{d_{g}\times d_{m}}), \end{aligned}\end{align*}

where $\mathcal{F}_{e}^{g}$ represents the multilayer perceptron network that maps the gene feature. After obtaining the respective representations of cells and genes, we proceed to execute the decoding process, which consists of two steps. The initial step entails the reconstruction of the cellular topological relationships. Adhering to the common practice in autoencoders, we extract features from the hidden layers and compute the inner product to obtain the reconstructed adjacency matrix: 


(9) \begin{align*}& \begin{aligned} \hat{\mathbf{A}}^{(c)}=\operatorname{sigmoid}\left(\mathbf{Z}^{(c)}*{\mathbf{Z}^{(c)}}^{\top}\right)\quad (\hat{\mathbf{A}}^{(c)} \in \mathbb{R}^{n \times n}), \end{aligned}\end{align*}

In addition, it is evident that the representations of cells $\mathbf{Z}^{(c)}$ and genes $\mathbf{Z}^{(g)}$ exist within the same feature space and are compressed to the $d_{m}$ dimension. Therefore, we calculate the matrix product of the acquired cell and gene representations to create the gene-cell cooperative embedding. 


(10) \begin{align*}& \begin{aligned} \hat{\mathbf{X}}^{(c)}=\left(\mathbf{Z}^{(c)}*{\mathbf{Z}^{(g)}}^{\top}\right)\quad (\hat{\mathbf{X}}^{(c)} \in \mathbb{R}^{n \times d_{g}}). \end{aligned}\end{align*}

It is important to note that the introduction of exogenous genes does not disclose cell identities. Incorporating gene embeddings should be regarded as feature mapping, reducing the original feature matrix from $d_{m}$ dimensions to a new feature space of $d_{g}$ dimensions through matrix multiplication. During the network training process, we promote consistency between the reconstructed cell graph matrix and the initial graph matrix, thus preserving the unified topological information. Meanwhile, the constructed gene-cell cooperative embedding is ensured to be consistent with the initial cell expression matrix. Mathematically, we minimize the mean squared error losses for both alignments as follows: 


(11) \begin{align*}& \begin{aligned} \mathcal{L}_{\mathrm{r}}=\left\|\mathbf{A}^{(c)}-\hat{\mathbf{A}}^{(c)}\right\|_{2}^{2} + \left\|\mathbf{X}^{(c)}-\hat{\mathbf{X}}^{(c)}\right\|_{2}^{2}. \end{aligned}\end{align*}

### Optimization of cell representation

The network that relies solely on reconstruction loss lacks adequate guidance during the training process, potentially leading to trivial solutions. Consequently, we introduce additional clustering losses to promote joint optimization. Although a variety of clustering losses are available, we will not delve into this aspect. This study focuses on the facilitative role that exogenous gene information plays in clustering. Nevertheless, this does not imply that this is the only available loss. We offer a paradigm, and readers are free to set an appropriate clustering loss. The detailed optimization process is described in Algorithm 1.

In this study, the widely used Kullback–Leibler (KL) divergence loss based on the Student’s $t$-distribution is employed as an example to guide network training. The formulation is as follows: 


(12) \begin{align*}& \mathcal{L}_{\mathrm{c}}=\sum_{i}\sum_{j} p_{i j} \log \frac{p_{i j}}{q_{i j}},\end{align*}

where the KL loss is employed to measure the difference between the computed clustering distribution $q_{ij}$ and the auxiliary target distribution $p_{ij}$. The $q_{ij}$ symbolizes the soft assignment in clustering, quantifying the degree of similarity between the latent layer of the $i$-th cell, denoted as $z_{i}$, and the $j$th cluster center, denoted by $\mu _{j}$. The computational process is outlined as follows: 


(13) \begin{align*}& q_{i j}=\frac{\left(1+\left\|z_{i}-\mu_{j}\right\|^{2}\right)^{-1}}{\sum_{j}\left(1+\left\|z_{i}-\mu_{j}\right\|^{2}\right)^{-1}},\end{align*}

subsequently, an auxiliary target distribution $p_{ij}$ was constructed based on the clustering distribution $q_{ij}$. 


(14) \begin{align*}& p_{i j}=\frac{q_{i j}^{2} / \sum_{i} q_{i j}}{\sum_{j}\left(q_{i j}^{2} / \sum_{i} q_{i j}\right)}.\end{align*}

The final complete loss function, which integrates both reconstruction and clustering losses, is expressed as follows: 


(15) \begin{align*}& \begin{aligned} \mathcal{L}=\mathcal{L}_{r}+\lambda\mathcal{L}_{c}, \end{aligned}\end{align*}

where $\lambda $ represents the hyperparameter that serves as a balancing factor between the reconstruction loss and the clustering loss.

### Generation of clustering results

The clustering result is derived from the refined cell embedding $\mathbf{Z}^{(c)}$. We utilize the k-means algorithm to obtain clustering outcomes for all samples. The k-means algorithm iteratively adjusts the cluster centers until they reach a stable state, minimizing the distance between each cluster center and its respective points. Transforming this clustering task into an optimization issue, the learned cell representation, $\mathbf{Z}^{(c)}$, is subjected to a factorization process, detailed as follows: 


(16) \begin{align*}& \begin{array}{l} \begin{array}{*{20}{c}} {\mathop{\min} \limits_{{\textbf{U,V}}}} &{{{\left\| {\mathbf{Z}^{(c)} - {\textbf{UV}}} \right\|}^{2}}}, \end{array}\\ s.t.{\textbf{U1}} = {\textbf{1}},{\textbf{U}} \ge{\textbf{0}}. \end{array}\end{align*}

Here, ${\textbf{U}} \in \mathbb{R}^{n \times k}$ represents the cluster indicator matrix, and ${\textbf{V}} \in \mathbb{R}^{k \times d}$ denotes the center matrix for clustering.

### Time complexity analysis

We have undertaken a simple analysis of the time complexity for the scEGG model, which aggregates to $\mathcal{O}(n^{2}d_{g}+d_{g}+nv+n d_{g}\tau )$, with $\tau $ signifying the count of training epochs. In detail, the construction of the KNN graph entails a computational expense of $O(n^{2}d_{g})$, whereas the generation of gene embeddings via node2vec demands $O(d_{g}+nv)$. The transformation of features within the neural network training process contributes to $O(n d_{g}\tau )$. It is apparent that, with an increase in the number of samples and the dimensionality of features, the algorithm will require a substantial amount of computational resources.

## Experiments

Extensive experiments were conducted to evaluate our model. For clarity, we assess the effectiveness of the proposed scEGG model by addressing the following research questions:

**RQ1.** How effective is the scEGG method in deep single-cell clustering tasks?
**RQ2.** Does the model learn clustering-friendly cell representations?
**RQ3.** What is the impact of exogenous genes on the performance of scEGG?
**RQ4.** What is the impact of hyper-parameters on the performance of scEGG?
**RQ5.** Does the scEGG model exhibit convergence?

### Experimental settings

#### Datasets and data preprocessing

This study employs six publicly available real-world datasets from common species such as humans and mice. The performance of clustering algorithms is assessed using external labels, with each dataset accompanied by its corresponding ground truth labels. The Darmanis [29] dataset includes human brain cells, which are noted for their complex composition and divided into multiple clusters. The Bjroklund [30] dataset comprises human lymphoid cells, which are crucial to the immune system. The Sun [31] dataset provides three single-cell datasets, however, our study focuses solely on the first, which contains exclusively mouse lung cells. Furthermore, the Marques [32] dataset investigates the developmental origins of oligodendrocyte precursor cells in mice. The Zeisel [33] dataset contains data from the somatosensory cortex and hippocampus CA1 regions of mice. Lastly, the Fink [34] dataset, sourced from the human adult ureter, could offer insights into metabolic processes.

**Figure 4:**
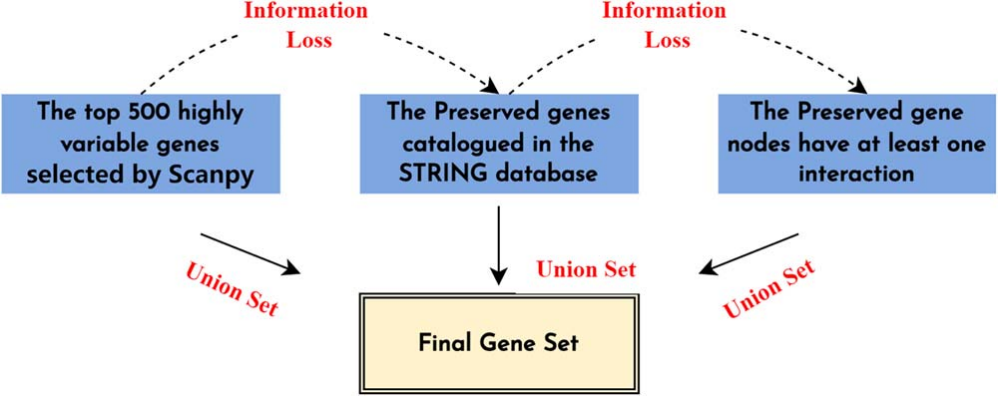
The gene selection process.

The initial scRNA data exhibit significant variability in scale and high noise levels, which could potentially lead to erroneous conclusions in subsequent analyses. To mitigate these issues, quality control was conducted on the cellular data prior to clustering. Specifically, cells with expression values within a reasonable range were retained, and outliers with extreme expression values were eliminated. This was achieved by establishing upper and lower thresholds at 75$\%$ plus three times the quartile deviation, and 25$\%$ minus the quartile deviation, respectively. Following quality control, the data were standardized by scaling to a consistent range. Subsequently, a log2 transformation was applied to the data. To avoid negative infinite values and ensure positive expression values, a pseudo count of 1 was incorporated during the transformation process. Additionally, gene selection was performed, retaining the highly variable genes using Scanpy. When duplicate gene names arise, it is recommended to average the expression values of the multiple genes to derive a consolidated expression value. The specific quantities are detailed in Table 1, and the gene selection process is presented in Fig. 4.

**Table 1:** Details of the seven real single-cell datasets. HVGs represent the highly variable genes selected via Scanpy

Datasets	Cells	Genes	HVGs	Clusters	References
Darmanis	290	9337	243	8	[29]
Bjorklund	647	26 087	326	4	[30]
Sun	1669	995	389	6	[31]
Marques	1854	15 291	357	14	[32]
Zeisel	2962	18 825	360	9	[33]
Fink	3030	20 932	385	7	[34]
Sun-PBMC	8197	1000	444	7	[31]

#### Benchmark methods

This section offers a concise overview of the baseline methods employed in the experiments. We introduced two classic single-cell clustering models, CIDR and Seurat, along with two deep clustering algorithms, DESC and scDeepCluster, and four recent comparative algorithms, scGAC, scDSC, scDFC, and scCAN.

(a) **CIDR** [11] utilizes a probabilistic model for evaluating dropout events in cellular data, categorized as a traditional clustering method in this study.
(b) **scDeepCluster** [18] introduces a deep autoencoder using the ZINB loss, classified as a deep clustering method in this research.
(c) **DESC** [17] employs an autoencoder network for cell embedding and batch effect elimination, distinguished as a deep clustering method in this research.
(d) **Seurat** [20] features a built-in Phenograph clustering method for constructing cell graphs via community detection, identified as a graph clustering method in this research.
(e) **scGAC** [23] introduces an attention mechanism in graph neural networks for efficient cellular graph construction, distinguished as a graph deep clustering method in this research.
(f) **scDFC** [24] merges cell attribute information with structural inter-cell information for clustering, recognized as a deep fusion clustering method in this study.
(g) **scDSC** [35] designs a deep structural clustering algorithm that incorporates structural information into the deep clustering of scRNA-seq data. By utilizing the ZINB model and graph neural network modules, it enhances clustering scalability.
(h) **scCAN** [36] proposes a novel imputation method based on adaptive neighborhood clustering to estimate dropout zeros in scRNA-seq data, thereby achieving superior clustering analysis.

#### Training details

The performance of the proposed algorithm was evaluated on an Ubuntu server featuring an Intel Core i9-12900KF CPU, 64GB of DDR4 memory, and an NVIDIA GeForce RTX 3070Ti graphics card. The Ubuntu system version is 22.04.2 LTS. The algorithm was implemented in Python 3.7, using the deep learning framework Pytorch version 1.13.1. The parameters of the node2vec algorithm were set to the default parameters. The bottleneck layer was set to 256. The model underwent training for 500 epochs. Learning rates were set at $10^{-5}$ for the training phase.

#### Evaluation

This study utilizes two widely used clustering evaluation metrics. Adjusted Rand Index (ARI) [37] measures the consistency between clustering results and true labels, necessitating labeled data. The formulation of this index is as follows: 


(17) \begin{align*}& \begin{aligned} \text{ARI} = \frac{\sum_{ij}\binom{n_{ij}}{2}-[\sum_{i}\binom{a_{i}}{2}\sum_{j}\binom{b_{j}}{2}]/\binom{n}{2}}{\frac{1}{2}[\sum_{i}\binom{a_{i}}{2}+\sum_{j}\binom{b_{j}}{2}]-[\sum_{i}\binom{a_{i}}{2}\sum_{j}\binom{b_{j}}{2}]/\binom{n}{2}}, \end{aligned}\end{align*}

where $a_{i}$ represents the number of samples in the $i$-th cluster of the true classification. $b_{j}$ denotes the number of samples in the $j$-th cluster of the algorithm-generated clustering results. $n_{ij}$ represents the number of consistent samples in both clustering results. Besides, Normalized Mutual Information (NMI) [38] assess the similarity between clustering results and true labels, requiring labeled data. The formulation of this index is as follows: 


(18) \begin{align*}& \begin{aligned} \text{NMI}=\frac{2 M I(U, V)}{H(U)+H(V)}, \end{aligned}\end{align*}

where $U$ and $V$ represent the clustering results of the ground truth labels and the algorithm-generated clustering results, respectively.

### Performance comparison (RQ1)

We executed a comprehensive series of experiments to compare the clustering performance of the proposed scEGG model and eight baseline methods. The findings unequivocally indicate that scEGG consistently achieved superior performance across all metrics, as detailed in Table 2. The top results are highlighted in red, while the runner-up is highlighted in blue. scEGG consistently ranked within the top two in all comparative analyses. Moreover, scEGG secured the best performance in 11 out of 14 comparisons, with improvements of (4.0%, 8.3%, 13.8%, 6.5%, 8.4%, 11.8%) on ARI and (14.3%, 8.9%, 0.3%, 8.9%, 3.5%) on NMI compared to the second place. The results shown in the table indicate that scDFC repeatedly secured the second place, illustrating the effectiveness of aggregating attribute information and structural information. The results of scCAN were relatively poor, which may be related to its design as a proprietary model for imputation rather than clustering. In summary, scEGG not only demonstrated its dominance in ARI but also exhibited significant stability in NMI.

**Table 2:** The ARI and NMI scores of scEGG and baseline methods across six datasets are presented (%)

	Datasets	Seurat	CIDR	scDeepCluster	DESC	scCAN	scGAC	scDFC	scDSC	scEGG
ARI	Darmanis	35.3	33.7	27.9	27.2	0.7	35.8	42.2	33.1	46.2 (4.0$\uparrow $)
	Bjorklund	5.6	45.7	31.0	41.2	0.2	72.9	43.2	2.2	81.2 (8.3$\uparrow $)
	Sun	18.2	26.8	78.3	60.3	4.9	32.1	78.6	33.7	92.4 (13.8$\uparrow $)
	Marques	17.2	10.0	39.0	26.9	0.6	26.6	42.7	16.8	49.2 (6.5$\uparrow $)
	Zeisel	11.9	16.7	50.4	31.0	0.2	29.3	53.4	50.6	61.8 (8.4$\uparrow $)
	Fink	6.3	22.5	35.9	21.2	0.4	48.5	45.1	32.2	60.3 (11.8$\uparrow $)
	Sun-PBMC	4.5	52.8	33.1	24.4	2.5	54.2	56.0	31.6	55.0 (1.0$\downarrow $)
**NMI**	Darmanis	62.2	55.4	46.6	54.0	5.9	30.8	51.2	47.3	57.2 (5.0$\downarrow $)
	Bjorklund	44.6	60.0	35.8	50.0	2.6	59.0	57.8	5.8	74.3 (14.3$\uparrow $)
	Sun	62.5	37.9	80.8	76.5	13.6	53.5	80.3	39.8	89.2 (8.9$\uparrow $)
	Marques	56.2	19.1	58.6	48.0	4.7	47.7	57.3	30.6	58.2 (0.4$\downarrow $)
	Zeisel	54.4	20.7	56.9	51.3	1.4	45.3	59.0	56.7	59.3 (0.3$\uparrow $)
	Fink	50.8	36.7	50.9	57.1	46.8	51.6	59.0	53.7	67.9 (8.9$\uparrow $)
	Sun-PBMC	43.2	55.6	51.4	50.8	4.1	59.4	59.6	29.1	63.1 (3.5$\uparrow $)

Table 3 displays the actual execution times of all algorithms, demonstrating that the runtime cost of scEGG is reasonable. Given the complexity of the biological environment, which contributes to the intricate distribution of single-cell data, identifying a universally applicable clustering method poses a considerable challenge. Nonetheless, scEGG displayed high performance in nearly all the evaluated tasks.

**Table 3:** The comparison of running times (s) of different algorithms

Datasets	Seurat	CIDR	scDeepCluster	DESC	scCAN	scGAC	scDFC	scDSC	scEGG
Darmanis	1.71	2.49	62.30	7.90	1.13	9.60	14.19	4.30	18.72
Bjorklund	5.15	16.80	4709.80	34.90	2.87	88.40	32.75	5.28	7.22
Sun	0.88	16.50	433.50	36.80	8.26	296.70	229.00	9.07	18.60
Marques	13.41	90.81	33.14	21.48	17.35	196.20	294.37	10.46	74.56
Zeisel	34.03	290.51	33.10	32.79	10.06	480.60	925.69	13.21	22.27
Fink	21.00	111.30	162.93	38.29	25.23	570.20	689.30	13.53	115.21
Sun-PBMC	6.03	1105.00	1879.00	98.00	100.29	6687.60	6615.21	40.48	95.30

### The investigation of cell representation (RQ2)

**Figure 5:**
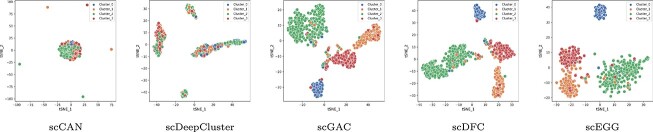
The two-dimensional t-SNE visualizations of cell embeddings on the Bjorklund dataset, learned under various comparative models.

The objective of representational learning is to acquire high-quality cellular embeddings, which directly influence clustering performance. To investigate whether the proposed scEGG model produces cluster-friendly cellular embeddings, we conducted a visual analysis using t-SNE on the embeddings generated by scEGG and other comparative models in the dataset Bjorklund, as displayed in Fig. 5. Specifically, cellular embeddings were obtained from the bottleneck layer of the models upon training completion. The experimental outcomes reveal that the embeddings from the scCAN model aggregated into a single cluster, lacking distinct dispersion among the four clusters, aligning with its low clustering performance, suggesting a poor quality of the learned cellular embeddings. In contrast, in scDeepCluster, although the cell populations were divided into four clusters, samples from different labels were intermixed within each cluster, highlighting a low accuracy in cell identity allocation. The cellular embeddings associated with scGAC and scDFC demonstrated an improvement in quality, yet adhesion at the edges persisted. The visual results of the scEGG model’s cellular embeddings exhibited clear separation between the four clusters, showcasing excellent intra-cluster cohesion and inter-cluster separation. This confirms that the scEGG model has successfully learned high-quality, cluster-friendly cellular representations.

### Ablation study (RQ3)

The core of this study is the introduction of exogenous gene information. To further illustrate the positive impact of the introduced information on clustering performance, we performed comprehensive ablation experiments. Specifically, we developed variants of scEGG by separately removing exogenous gene information and the constructed clustering loss, as well as removing and retaining both simultaneously, resulting in four variants. We compared the clustering performance of these four variants in Table 4, and the results show that scEGG, which retains both modules, exhibits superior clustering performance.

**Table 4:** The ablation study utilizing ARI and NMI metrics: we successively removed the exogenous gene module and clustering loss from the model to observe the respective impacts on performance. $E\_G$ represents the exogenous gene, while $\mathcal{L}_{c}$ denotes the clustering loss (%)

Module		ARI					
$\boldsymbol{ E\_G}$	$\boldsymbol{\mathcal{L}_{c}}$	Darmanis	Bjorklund	Sun	Marques	Zeisel	Fink
$\times $	$\times $	31.7	61.9	77.9	46.9	59.2	54.8
$\times $	$\checkmark $	44.6	81.0	89.0	48.2	61.4	58.0
$\checkmark $	$\times $	44.0	80.0	86.0	46.5	51.7	57.0
$\checkmark $	$\checkmark $	46.2	81.2	92.4	49.2	61.8	60.3
		**NMI**					
$E\_G$	$\mathcal{L}_{c}$	Darmanis	Bjorklund	Sun	Marques	Zeisel	Fink
$\times $	$\times $	48.6	63.2	79.3	56.8	56.6	65.5
$\times $	$\checkmark $	54.8	74.0	86.0	57.9	59.0	66.2
$\checkmark $	$\times $	54.9	73.2	82.9	56.3	49.8	66.0
$\checkmark $	$\checkmark $	57.2	74.3	89.2	58.2	59.3	67.9

**Table 5:** The parameter analysis utilizing ARI and NMI values: It involved iterating over the parameter set $\{1, 10^{1}, 10^{2}, 10^{3}, 10^{4}\}$ to investigate the impact of the hyperparameter $\lambda $ on the model’s clustering performance (%)

Metric	Datasets	$\boldsymbol{\lambda =1}$	$\boldsymbol{\lambda =10^{1}}$	$\boldsymbol{\lambda =10^{2}}$	$\boldsymbol{\lambda =10^{3}}$	$\boldsymbol{\lambda =10^{4}}$
**ARI**	Darmanis	44.6	44.6	45.1	46.2	44.6
	Bjorklund	81.0	81.0	81.2	81.7	81.3
	Sun	89.0	92.7	92.4	92.0	90.9
	Marques	48.2	49.0	49.2	48.0	45.7
	Zeisel	61.6	61.3	61.8	60.3	57.7
	Fink	58.0	58.0	60.3	57.9	54.6
**NMI**	Darmanis	54.8	54.8	55.1	57.2	54.7
	Bjorklund	74.0	74.0	74.3	74.8	74.6
	Sun	86.0	89.6	89.2	88.6	87.4
	Marques	57.9	58.3	58.2	57.7	57.2
	Zeisel	59.3	58.9	59.3	58.1	56.0
	Fink	66.2	66.2	67.9	66.0	62.5

Removing the exogenous gene module leads to a significant performance decline, emphasizing the insightful and critical role of exogenous genes in clustering. While removing the clustering loss does not cause a dramatic decline in clustering performance, a decrease is still observed, indicating that without clustering loss, the network’s training loses guidance. The integration of both modules yields the best performance, demonstrating that the constructed components have a significant impact on the model. Removing both modules simultaneously resulted in the worst clustering performance, which is consistent with our expectations. In summary, the ablation experiments conclude that appropriately incorporating exogenous gene information into the neural network training process helps generate discriminative cell representations, leading to superior clustering performance.

### Parameter sensitivity analysis (RQ4)

The scEGG model simultaneously introduced the reconstruction loss and the clustering loss to guide the training of the neural network. To balance the reconstruction loss and the clustering loss, a weighting coefficient represented by $\lambda $ is introduced in Equation (15). This section provides an in-depth analysis of this hyperparameter and its impact on clustering outcomes. To determine the optimal weight allocation, experiments were conducted across six datasets. Specifically, for each dataset, the parameter set $\{1, 10^{1}, 10^{2}, 10^{3}, 10^{4}\}$ was iterated, and the corresponding clustering performance was recorded in Table 5, with the best and second-best performers highlighted in red and blue, respectively. The results from the table indicate that the overall model performance exhibits minimal variation with changes in the this parameter, suggesting that the model is not highly sensitive to $\lambda $ adjustments. However, minor fluctuations were observed as this parameter varied, with $\lambda =10^{2}$ achieving the highest frequency of top two positions. Therefore, it is recommended to set the hyperparameter to $10^{2}$ for optimal clustering performance. Exploring the parameter space is essential to achieve optimal results for each dataset.

**Figure 6:**
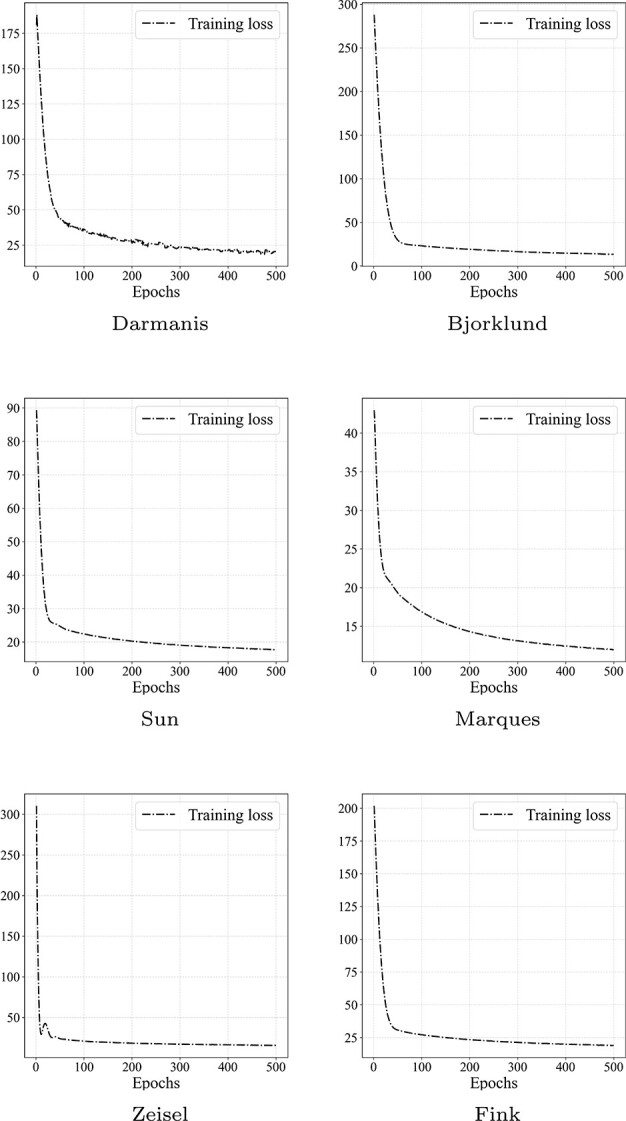
The objective function was recorded over 500 epochs during the training on six benchmark datasets.

### Convergence analysis (RQ5)

Convergence is a vital aspect of assessing model performance. In this section, the convergence of the scEGG model is investigated. Specifically, the loss values at each epoch are monitored to determine whether they decrease and ultimately stabilize at a constant value. To provide a clearer visual representation, a curve of the loss values has been plotted in Fig. 6. The result clearly demonstrates that, across all datasets, the loss values progressively decrease and ultimately converge to a fixed value. Despite varying convergence rates across different datasets, such as rapid convergence on the Zeisel dataset and noticeably slower convergence on the Marques dataset. These findings substantiate the scEGG model’s convergence.

### Investigating risks associated with the introduction of exogenous information

Exogenous information provides comprehensive insights into single-cell data. However, incorporating exogenous information into a deep learning framework must be approached with caution, as its introduction can also pose risks. In this section, we examine the risks associated with the introduction of exogenous information and discuss strategies to mitigate these risks.

The primary risk concerns whether the exogenous information will introduce knowledge bias, potentially causing the model to depend on patterns within this exogenous information in ways that are not immediately transparent, thus steering the model’s clustering decisions toward pre-existing knowledge.

However, nearly all unsupervised clustering algorithms exhibit this type of knowledge bias. The clustering process assigns each sample to a cluster based on the Euclidean distance in a high-dimensional space. This knowledge bias can cause samples with smaller Euclidean distances to cluster together, even if they are distant in the real world. Although unsupervised clustering algorithms do not require external labels, they still operate based on specific computational rules. A critical aspect is whether the computational rules disclose the identity of the cell. Clearly, scEGG relies on an existing feature knowledge base to perform a fixed transformation of the original features, without revealing the cell’s identity. From a macro perspective, this process remains unsupervised, as the feature is continuously mapped to a higher dimension, and the ultimate result still depends on the Euclidean distance in a specific high-dimensional space.

**Figure 7:**
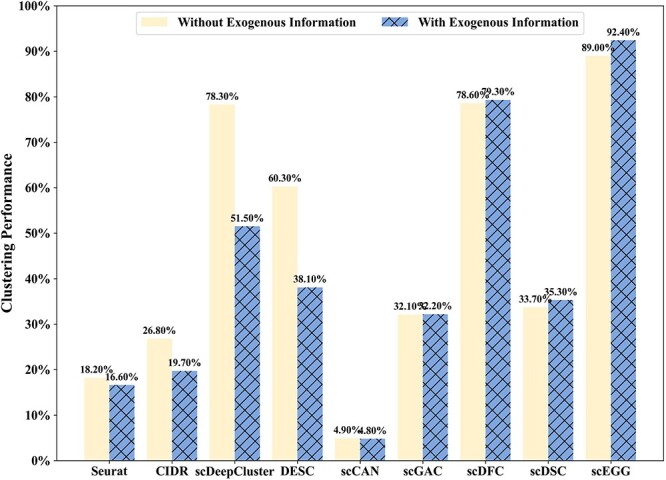
The investigation assesses the impact of exogenous information on the clustering performance across nine models on the Sun dataset.

To further validate this point, we have supplemented our study with additional ablation experiments focusing on exogenous information, the findings of which are detailed in Fig. 7. Our findings indicate that the introduction of exogenous information does not consistently benefit clustering, potentially enhancing or impairing the performance of various models. This lends support to our hypothesis that the incorporation of exogenous information remains an unsupervised process.

Another risk associated with the introduction of exogenous information is the potential for feature truncation, which could lead to NaN values when this information is incorporated into certain models. This issue emerges because the encoding of genes and cells is not calculated in the same manner, potentially causing value overflows during matrix transformations. Despite this, the scEGG model maintains stable performance through its well-designed joint training module, which fine-tunes cell embeddings and preserves most information from prior learning phases in each new iteration, suggesting that fine-tuning might represent a viable strategy to mitigate this risk. Additionally, monitoring numerical values in deep networks could effectively pinpoint the locations where feature truncation occurs within the model.

## Conclusion

In conclusion, we have developed scEGG, an effective exogenous gene-guided clustering model for single-cell data that creates gene-cell cooperative embeddings to promote clustering. Although streamlined in architecture, it provides a paradigm for introducing exogenous medical information into the process of deep clustering and has the potential to be expanded to include more functions. The specially designed reconstruction and clustering optimization module enhances the quality of cell embeddings while maintaining the cell’s topological structure during the optimization process. Our experimental findings demonstrate that exogenous genes significantly improve embedding optimization, and our model surpasses other existing methods.

While effective, the feature mapping associated with the exogenous gene module is not universally applicable to all clustering models at this stage. Direct incorporation of the exogenous gene matrix into the current clustering framework may cause feature truncation. The universality of the exogenous gene module requires further development. In the future, we aim to investigate innovative approaches to random walks on the gene graph for more effective representations. Additionally, we plan to examine more similarity measures of cells to construct a more precise cell graph [39–44]. Collaborative training presents another exciting research direction, as we believe that integrating cell and gene data can mutually enhance clustering effectiveness [45, 46].

**Key Points**

- We pioneered an exogenous gene-guided clustering framework that generates gene-cell cooperative embeddings and learns a more discriminative representation through optimization. This work establishes a paradigm for integrating exogenous medical information into the clustering process.
- The proposed scEGG model employs a GAT to accurately aggregate information among cells and uses reconstruction loss and clustering loss to facilitate the optimization of the bottleneck layer, effectively utilizing its own information without requiring labels.
- The experiment demonstrates the effectiveness and superior performance of scEGG when compared to the other eight baseline methods.

## Data Availability

The datasets and code can be publicly accessed in Repository https://github.com/DayuHuu/scEGG.
